# Homogeneous InGaSb crystal grown under microgravity using Chinese recovery satellite SJ-10

**DOI:** 10.1038/s41526-019-0068-1

**Published:** 2019-04-01

**Authors:** Jianding Yu, Yuko Inatomi, Velu Nirmal Kumar, Yasuhiro Hayakawa, Yasunori Okano, Mukannan Arivanandhan, Yoshimi Momose, Xiuhong Pan, Yan Liu, Xingwang Zhang, Xinghong Luo

**Affiliations:** 1 State Key Laboratory of High Performance Ceramics and Superfine Microstructure, Shanghai Institute of Ceramics, Chinese Academy of Sciences, 1295 Dingxi Road, Shanghai, 200050 PR China; 2 Centre of Materials Science and Optoelectronics Engineering, University of Chinese Academy of Sciences, Beijing, 100049 PR China; 3 Institute of Space and Astronautical Science, Japan Aerospace Exploration Agency, 3-1-1 Yoshinodai, Chuo-ku, Sagamihara, Kanagawa 252-5210 Japan; 4 School of Physical Sciences, SOKENDAI (The Graduate University for Advanced Studies), 3-1-1 Yoshinodai, Chuo-ku, Sagamihara, Kanagawa 252-5210 Japan; 5 Research Institute of Electronics, Shizuoka University, Johoku 3-5-1, Naka-ku, Hamamatsu, Shizuoka 432-8011 Japan; 6 Graduate School of Engineering Science, Osaka University, 1-3 Machikaneyama, Toyonaka, Osaka 560-8531 Japan; 7 Centre for Nanoscience and Technology, Anna University, Chennai, 600025 Tamil Nadu India; 8 Institute of Semiconductors, Chinese Academy of Sciences, No. A35, Qinghua East Road, Haidian District, Beijing, 100083 PR China; 9 Institute of Metal Research, Chinese Academy of Sciences, 72 Wenhua Road, Shenyang, Liaoning 110016 PR China

## Abstract

Microgravity crystal growth experiment for the growth of In_0.11_Ga_0.89_Sb was performed at the Chinese recoverable satellite through the space program SJ-10. This experiment is aimed to understand the melt formation and growth kinetics of In_*x*_Ga_1−*x*_Sb solid solution with higher indium composition, because their segregation coefficient was higher than the crystals with lower indium compositions. The target composition and uniformity were achieved with higher growth rate under microgravity, whereas the uniformity in composition was not achieved under normal gravity. The growth and dissolution were affected mainly by the steady state equilibrium in the melt composition because of the convection under normal gravity. The non-steady state equilibrium in the melt composition under microgravity helped to achieve a higher growth rate and compositional homogeneity at higher indium composition of In_*x*_Ga_1−*x*_Sb solid solution.

## Introduction

Thermophotovoltaic (TPV) devices convert thermal radiation from an emitter into electricity which is composed of an emitter, a filter, and a photovoltaic (PV) cell. In the TPV devices, the distance between the emitter and the photoelectric conversion element is close to a few centimeters. Therefore, the energy density incident on the photoelectric conversion element is as high as 1 W/cm^2^, and an incident energy density with 100 times that of the Sun is obtained. The theoretical value of the conversion efficiency (of the TPV device) is about 30%.^1^ Two kinds of emitters are used in the TPV devices. One is a non-selective emitter such as SiC, and the other is a selective emitter such as rare-earth doped Al_2_O_3_. When the holmium-doped Al_2_O_3_ emitter is used, the emitted wavelength is 2 μm. For an efficient TPV device, the wavelength of the emitter and the absorption wavelength (band gap) of the PV cell should be adjusted. The absorption wavelength and lattice constant of In_*x*_Ga_1−*x*_Sb can be controlled in the range of 1.7–6.8 μm and 6.096–6.479 Å by adjusting the indium composition, respectively. It was calculated that In_0.11_Ga_0.89_Sb has the wavelength of 2 μm and hence it can be a potential material as the PV cell to fabricate the TPV device with Al_2_O_3_ emitter.

The growth of high-quality homogeneous In_*x*_Ga_1−*x*_Sb bulk crystals is a challenging task because there is a large separation between the solidus and liquidus lines in the InSb–GaSb binary phase diagram.^2^ Since the composition in the crystal is different from that in the solution, the rejected solute accumulates near the growth interface. As a result, the composition in the crystal varies along the growth direction. Moreover, the segregation and temperature fluctuation by buoyancy convection under terrestrial condition could cause solid/liquid (S/L) interface breakdown and consequently polycrystalline crystals are grown.^3^ Since the transportation of the solute in the melt is affected by the convection, the dissolution and growth processes are strongly influenced by gravity.^4–7^

Microgravity is an excellent condition to suppress the convection, and complexed heat and mass transports to make deeper insight into the solute transport phenomena and understand the growth process of ternary alloys. GaSb growth experiments were carried out under microgravity conditions in space.^8–12^ Hayakawa et al. performed several microgravity experiments using a space shuttle, a drop tower, a recoverable satellite, and an airplane in the past 2 decades.^13–18^ These experiments were designed to reveal a distinct phenomenon in a step-by-step sequence. For instance, the effects of melt mixing among the elements with different densities were done in 1994 during IML-2 mission (Second International Microgravity Laboratories) using a Sb/GaSb/In sandwich system. It was revealed that the melt mixing was controlled by diffusion, and Marangoni convection occurred due to the free surface area of melt.^13,14^ In an experiment (conducted during 1996) at the Chinese recoverable satellite (CRS), a GaSb(111)A/InSb/GaSb(111)B system was used in a horizontal configuration to observe crystallization from (111)A and (111)B planes of GaSb, simultaneously.^16^ This experiment was conducted for about 4 h with a temperature profile that was decreased gradually without any holding process, because of its short duration. A parallel solid–liquid interface and uniformity in composition under microgravity were revealed in that experiment.^16^

Similarly, experiments were conducted under reduced gravity conditions using the drop tower and parabolic flight to observe the recrystallization processes using the same In–Ga–Sb system.^17,18^ These microgravity experiments were conducted for a short duration of about 5 s to 4 h. The results showed that for observing major crystal growth phenomena such as melt formation, dissolution, solute transport, diffusion, nucleation, growth, etc., a long duration for about a week is required. Additionally, the higher indium composition of InGaSb led to the formation of cracks due to compositional segregations. To overcome these issues, a sequence of four distinct experiments were designed and done under prolonged microgravity for about 240 h on board the International Space Station (ISS) through “Alloy Semiconductor Project”.^19–21^ These experiments were focused to observe the orientation-dependent dissolution and crystallization of InGaSb through the primary planes of GaSb (111)A, (111)B, (110), and (100). The lower indium composition (0.03) was selected for these experiments because the orientation-dependent growth process is more sensitive for segregations at higher indium compositions.

As the growth of In_*x*_Ga_1−*x*_Sb with higher indium composition is becoming more complicated because of their large segregation coefficients, their solute transport and growth mechanism will largely differ from the growth process of In_*x*_Ga_1−*x*_Sb with low indium compositions. Because of this segregation effect, the homogeneous composition of In_*x*_Ga_1−*x*_Sb at higher indium composition was not achieved under normal gravity. Additionally, the behavior of immiscible In–Ga–Sb system with higher indium composition is unclear yet.

Thus, considering the importance and possibilities to grow In_*x*_Ga_1−*x*_Sb with higher indium composition, another microgravity experiment was designed and conducted in 2016, to grow In_0.11_Ga_0.89_Sb crystal using a CRS for a long duration of 62 h, through the space program SJ-10.^18,24^ Though recoverable satellites could provide microgravity condition for about a week, due to the schedule for other experiments, our experiment was conducted for a maximum duration of about 62 h. SJ-10 recoverable scientific experimental satellite was launched by the Long March 2D carrier rocket in Jiuquan Satellite Launch Centre of China on April 6, 2016, and recovered on April 18, 2016, successfully. SJ-10 carried out the missions of space microgravity experiments in both fields of physical science and life science at low Earth orbit for 2 weeks.^22–25^ A multi-function furnace for eight materials research mission was installed in the satellite. Shanghai Institute of Ceramics, Chinese Academy of Sciences provided experimental data and a part of the space-grown sample acquired through the SJ-10 mission to Japanese science team members for the analysis.

In this manuscript, we report our microgravity experiment using CRS (SJ-10 space program) to grow In_0.11_Ga_0.89_Sb crystals with a homogeneous composition which is not achieved yet on the ground, successfully. The experimental procedures, compositions, and growth kinetics of In_0.11_Ga_0.89_Sb crystals under μG in comparison with a ground experiment are explained.

## Results

**Fig. 1:**
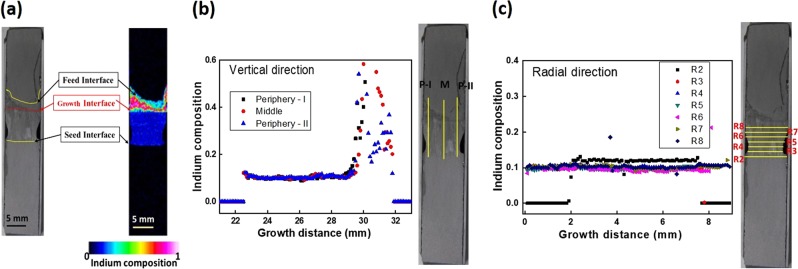
Indium composition of In_0.11_Ga_0.89_Sb under microgravity. **a** EPMA mapping, **b** along vertical direction, and **c** along radial direction (growth region)

The indium composition of the space-grown crystal measured by EPMA is shown in Fig. 1a, which is the schematic representation of the dissolved area and compositional mapping of the crystal. From the results, it was observed that In_0.11_Ga_0.89_Sb crystal with a length of about 7.1 mm was grown. The indium composition at the GaSb seed interface was 0.11, which corresponded to the growth temperature of 643 °C. As the In_0.11_Ga_0.89_Sb crystal was grown to be 7.1 mm during 49 h, the average growth rate was calculated to be 0.145 mm/h. The indium composition along the longitudinal and radial positions is shown in Fig. 1b, c. The dissolution length of the seed and feed crystals was also calculated from the EPMA data. It was found that 0.5 and 4.8 mm of seed and feed crystals were dissolved during the experiment.

**Fig. 2:**
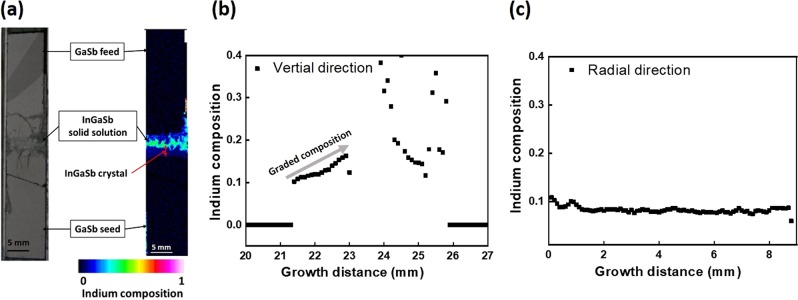
Indium composition of In_*x*_Ga_1−*x*_Sb under normal gravity. **a** EPMA mapping, **b** along vertical direction, and **c** along radial direction (growth region)

Similar growth experiment was performed under normal gravity condition on Earth. The EPMA mapping and indium composition along the vertical and radial directions are shown in Fig. 2a–c. The total length of the grown crystal was lower to be 1.5 mm with its indium composition varying from 0.10 to 0.16. The average growth rate was estimated to be 0.03 mm/h. From the undissolved seed and feed crystals, it was found that 1.7 mm of seed and 1.1 mm of feed crystals were dissolved.

The dissolution lengths of seed and feed crystals under microgravity and normal gravity conditions are compared and given in Table 1. The seed crystal dissolution was higher under normal gravity, and the feed crystal was dissolved more under microgravity. Because the feed crystal dissolution was very less, the growth rate was observed to be low under normal gravity.

**Table 1:** Dissolution lengths of seed and feed crystals

Condition	Crystal interface	Distance (mm)		Dissolution length (mm)
		Initial	Final	
Microgravity	Seed	23	22.5	0.5
	Feed	27	31.8	4.8
Normal gravity	Seed	23	21.3	1.7
	Feed	27	28.1	1.1

## Discussion

The shape of the growth interface was almost parallel at the onset and end of the growth, and the indium composition was uniform under microgravity. The length of the grown crystal was less than the InGaSb crystal grown at the ISS because the duration of ISS experiment was longer to be 210 h. The indium composition along the growth direction (Fig. 1b) was a little high at the initial and final stages of the growth. It might be because of the growth of crystal during heating and cooling processes. The indium composition was uniform to be around 0.11 from the distance 22.7–29.2 mm, that indicated the growth interface temperature was maintained constant around 643 °C. At the initial and final stages of the growth, the indium composition was a little higher (up to 0.15) because of the growth at the “end of heating” and “beginning of cooling” processes. During the cooling process, the indium composition was gradually increased, and when the cooling rate becomes more, the remaining indium-rich melt was solidified at the final stage of growth. The uniform composition along the radial direction (Fig. 1c) shows that the temperature distribution was uniform across the melt during the growth process. The feed crystal was dissolved more under microgravity because of its contact with the melt throughout the growth process. Whereas the dissolution of seed interface was stopped entirely because it became the growth interface after the nucleation process.

The indium composition under normal gravity was gradually increased along the growth direction (Fig. 2b). The increase of indium composition indicated that the temperature at the growth interface was decreased during the growth process. The cooling rate (0.51 °C/mm) to replicate the space experiment was not successful under normal gravity. It was higher than the actual growth rate for maintaining the uniform composition at the growth interface. This resulted in the increase of indium composition along the growth direction with decreased temperature.

At a constant growth interface temperature (both under microgravity and normal gravity), to grow InGaSb with the indium composition 0.11 (solidus composition), the equilibrium liquidus composition should be 0.5, according to the phase diagram values. Because indium is readily available in the InSb melt, GaSb with an equal amount (0.5) should be dissolved to reach an equilibrium of its composition in the liquid state. For the In_0.11_Ga_0.89_Sb crystal to grow, appropriate compositions of In (0.1) and Ga (0.9) should be incorporated at the growth interface either by the dissolution of more GaSb molecules or ejection of excess indium into the melt. The seed crystal was dissolved more under convective flow (normal gravity) condition. It indicated that the growth was initiated by the dissolution of more GaSb molecules rather than ejection or diffusion of indium into the melt, under normal gravity condition. Whereas under microgravity, the seed crystal dissolution was low (this was similar to that of the ISS experiments) that indicated excess indium might eject into the melt by the diffusion process.

These experimental results suggest that the composition of the melt was saturated, and a state close to equilibrium was achieved earlier under normal gravity. Moreover, the equilibrium (compositional) under normal gravity became a steady state phenomenon because of the convection process. Once the melt composition was reaching close to the equilibrium-steady state, the dissolution from the dissolution front (feed crystal) was suppressed, and the growth rate was reduced. Whereas under microgravity, the convection was absent and the dissolution of GaSb feed crystal was diffusion controlled. As this VGF method involves the application of temperature gradient, the equilibrium-steady state in melt composition could not be achieved under microgravity. Though the seed interface was supersaturated, and the growth was initiated from the seed crystal, the compositional equilibrium under microgravity is a non-steady state phenomenon which could last longer until the temperature becomes uniform. Thus, the feed crystal was dissolved continuously during the holding process, and the length of the grown crystals was long under microgravity than that of under normal gravity. The results were similar to the long duration microgravity experiments performed at the ISS and on Earth.^19^ Based on the experimental results (in consideration with earlier experiments at the ISS), we may conclude that normal gravity is useful to achieve a steady state of equilibrium in the composition of the melt. However, the non-steady state of equilibrium in the melt composition under microgravity could be helpful for better dissolution and crystallization kinetics that would result in a higher growth rate.

As a conclusion, In_0.11_Ga_0.89_Sb with uniform composition was grown for the first time under microgravity environment on board CRS SJ-10. The shapes of initial and final growth interfaces, the dissolution tendency of the seed and feed crystals, and growth kinetics of this CRS experiment were similar to the long duration microgravity experiments performed at the ISS. This provides an evidence for the repeatability and reproducibility of the microgravity experimental results. The target composition and uniformity were achieved under microgravity and they were not achieved under normal gravity because of its lower growth rate. Normal gravity is useful to achieve a steady state of equilibrium in the composition of melt. However, the non-steady state of equilibrium in the melt composition under microgravity could be helpful for better dissolution and crystallization kinetics that resulted in higher growth rate.

## Methods

**Fig. 3:**
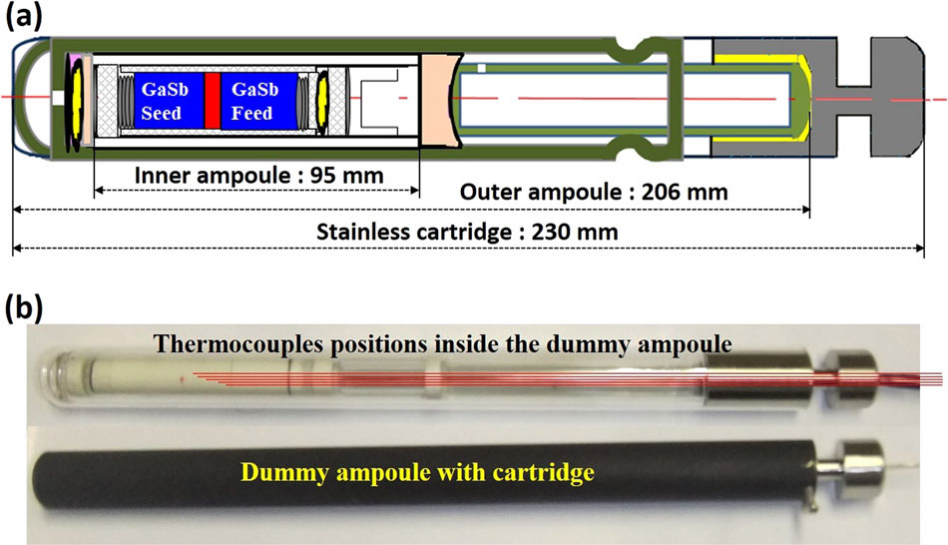
**a** The schematic of the ampoule for the growth of In_0.11_Ga_0.89_Sb. **b** A set of five fine K-type thermocouples within a dummy ampoule for the temperature calibration

In_0.11_Ga_0.89_Sb crystal was grown under microgravity at the CRS by a vertical gradient freezing method. An ampoule was specially designed to fit into the standard furnace that was installed at the CRS. The schematic of the ampoule prepared with GaSb(111)A/InSb/GaSb(111)A crystals for the growth of In_0.11_Ga_0.89_Sb is shown in Fig. 3a. GaSb (111)A single crystals grown by the Czochralski method and InSb crystals were cut and polished into a cylindrical shape to prepare the ampoule.^26–28^ The crystals were polished by alumina abrasive to get a mirror surface and etched in a mixture of HF:HNO_3_:CH_3_COOH (1:1:1) to remove the oxide layers from the surface. The lengths of GaSb and InSb crystals were 23 and 4 mm, respectively, and the diameter was 9 mm. A GaSb(111)A/InSb/GaSb(111)A sandwich sample was inserted into a BN tube that was kept inside a quartz ampoule under nitrogen flowing atmosphere. Carbon sheets baked at 800 °C were adopted on either side of the ampoule to adjust the volume change during the dissolution and growth processes. The ampoule was evacuated to 10^−4^ Pa and sealed off. This inner ampoule has 13 mm *ϕ* and 122 mm length. The inner ampoule was packed in another quartz tube to make an outer ampoule, enabling to adjust its length and dimensions to be fit with the furnace cartridge. The ends of the inner ampoule were packed with quartz wool and carbon sheets to prevent accidental breaking of the ampoule.

**Fig. 4:**
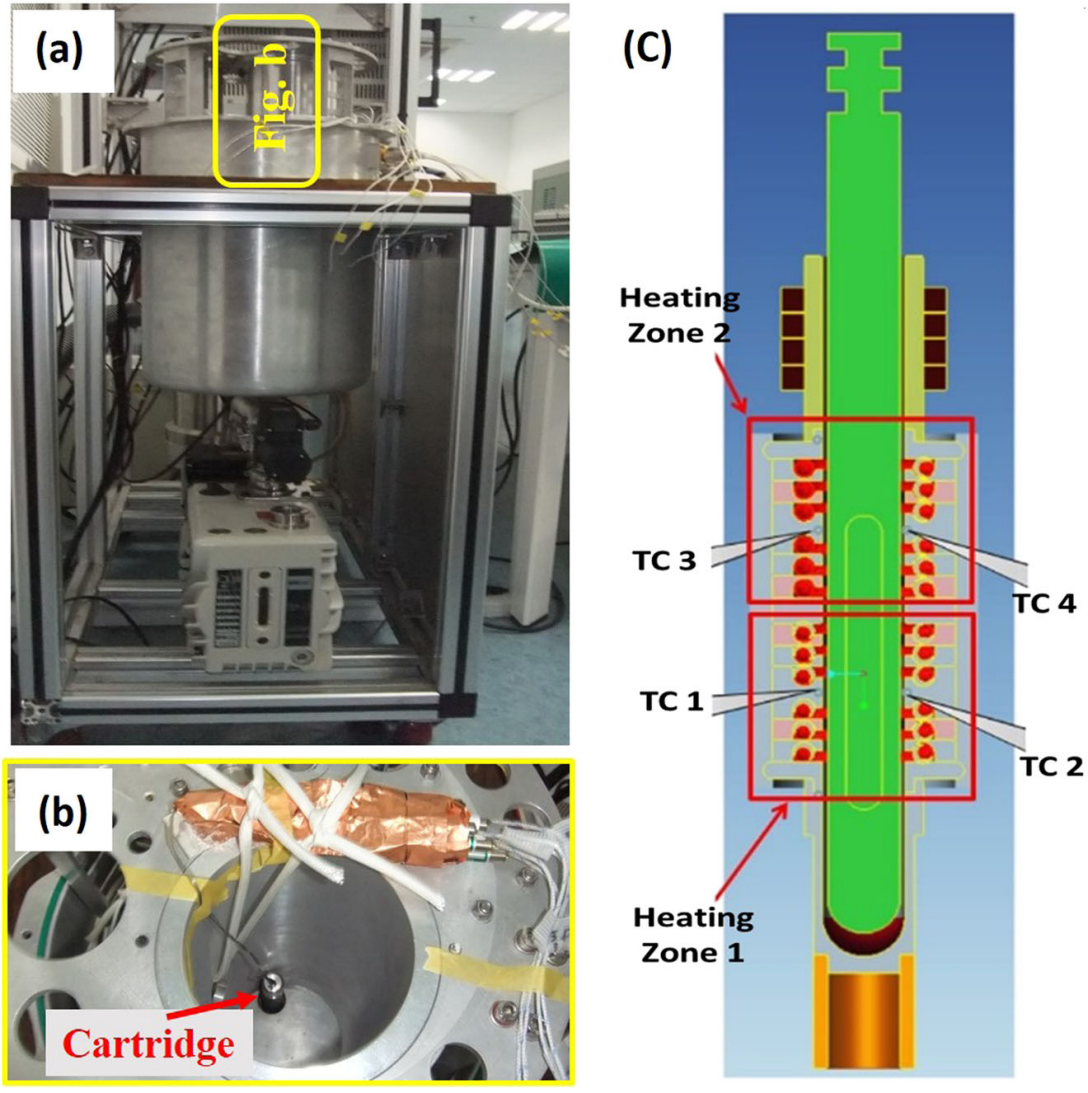
**a** Ground furnace for the temperature calibration. **b** Top view of the furnace consisting the dummy ampoule for temperature measurement. **c** A schematic of the furnace configuration and position of the cartridge

To calibrate the furnace based on the temperature inside the cartridge, a dummy ampoule with a BN sample was designed and prepared as shown in Fig. 3b. Five fine K-type thermocouples with a diameter of 0.1 mm were set into the position of the growth interface region and the dummy ampoule within a stainless cartridge was used to measure the temperature distribution in the sample mounting region. The furnace used for temperature calibration is shown in Fig. 4a. The dummy ampoule was placed inside the furnace (Fig. 4b) and a temperature profile was applied to it. The temperature of the five thermocouples (inside the dummy ampoule, at the growth region) were recorded, simultaneously. Based on the recorded temperature profiles the set temperature of the furnace was fixed to be 705 °C.

The schematic of the furnace configuration is shown in Fig. 4c. The furnace had a two-zone configuration to maintain a high-temperature gradient at its core. Thermocouples 1 and 3 were used to measure the temperatures of zones 1 and 2, respectively. The thermocouples 2 and 4 were backup parts in case the thermocouples 1 and/or 3 could not function well. From several measurements, it was confirmed that the temperature of the heating zone 1 had a 30–40 °C higher temperature than the sample mounting region. This temperature difference between inside and outside of the cartridge was resulting from the heat loss according to the heating rate, dissipation, and vacuum level. The target temperature of the furnace was fixed considering the temperature difference between inside and outside of the cartridge, and the growth temperature of In_0.11_Ga_0.89_Sb crystal.

The composition of the In_*x*_Ga_1−*x*_Sb crystal depends on the temperature of the growth interface. The InSb–GaSb binary phase diagram was used to fix the growth temperature to be 643 °C to grow In_0.11_Ga_0.89_Sb.^29^ Because the time duration to perform growth experiments at recoverable satellites are limited, unlike the long duration experiments at the ISS, the temperature program to grow In_0.11_Ga_0.89_Sb at CRS was restricted with the limited duration of 62 h. Initially, the furnace was heated from RT to the growth temperature at 3 h and kept hold for 3 h to proceed with the dissolution process. During this holding time, InSb crystal melted at 525 °C and GaSb seed and feed crystals started to dissolve in the InSb melt to make In_*x*_Ga_1−*x*_Sb solution. Since the temperature gradient was maintained, the temperature of feed interface was higher than seed interface. A compositional gradient was established by the variations in the dissolution of GaSb seed and feed crystals because of the temperature gradient of the furnace. When the melt composition tends to attain equilibrium, the low-temperature seed interface got supersaturated, and crystal started to grow from seed interface without moving the ampoule. The dissolution of the feed crystal continuously assisted the growth of the crystal. After holding for 3 h at the growth temperature, the temperature was decreased at a rate of 0.5 °C/h to grow homogeneous crystals for 49 h. It is necessary to apply such a cooling rate at the holding temperature to grow homogeneous crystal because the temperature of growth interface was increased according to the temperature gradient in the solution, which could cause a gradual decrease of indium composition in the grown crystal.

**Fig. 5:**
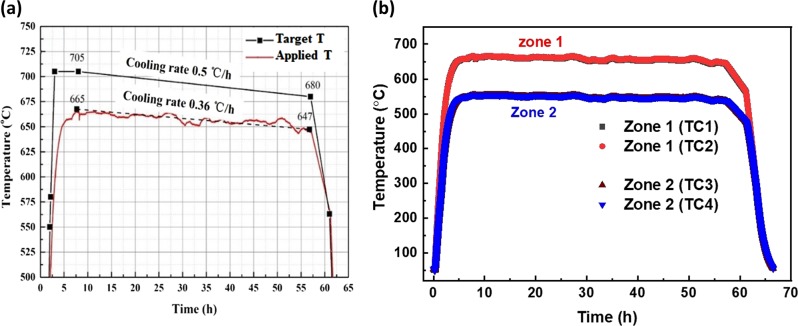
Temperature profiles (microgravity): **a** the target and applied temperatures to the furnace and **b** temperatures measured by four thermocouples in the zones 1 and 2 of the furnace, during the experiment

Figure 5a shows the temperature profiles of target and applied temperatures to the furnace for running the experiments. The furnace temperature can be controlled and recorded by tele-operations in real time by the ground control station.^30^ The target temperature 705 °C was fixed initially, so as to reach the growth temperature 643 °C for growing In_0.11_Ga_0.89_Sb crystal, based on the ground experiments.

However, the target temperature was not applied to the furnace because of some trouble in the battery system that gives power supply to the furnace heaters. Due to the failure of automatic power supply, the furnace power was controlled manually from the ground station. With the availed limited power, the furnace temperature reached only up to 665 °C. Figure 5b shows the temperature profiles of zones 1 and 2 recorded during the experiment. It revealed that though the applied temperature (665 °C) of the furnace was lower than the target temperature (705 °C), the temperature of zone 1 was not affected and it affected only the temperature of zone 2. As the ampoule was kept in zone 1, the growth region was not affected by this unexpected failure of the furnace power. The zone 1 temperature was recorded to be 660 °C (Fig. 5b), and it should be noted that the actual temperature inside the ampoule (at the growth interface) is unknown. However, the actual temperature at the growth interface could be identified by comparing the indium compositions of the grown crystal with the GaSb–InSb binary phase diagram values.

An experiment was conducted on ground using a 3-zone vertical gradient furnace (VGF) in Japan, to replicate the microgravity experiment at the CRS. The furnace installed at CRS is a specially designed one with an automatic cartridge loading mechanism (using a barrel system which comprising eight cartridges for the experiments by various groups). That multi-function furnace was not available for conducting the ground-based experiment for all the research groups and thus we used our 3-zone VGF in Japan that was similar to the furnace installed at the ISS. The temperature of each zones in the VGF could be independently controlled to maintain uniform temperature distribution inside the cartridge. The cartridge was specially designed based on numerical simulations and it can maintain a uniform temperature in the growth region. The furnace set temperature and duration for the ground experiment were 655 °C and 65 h, respectively. The set temperature for the VGF furnace is fixed based on the previous experiments to grow In_0.11_Ga_0.89_Sb crystals. The detailed experimental procedures using the VGF are explained in our previous reports.^19,20^

The total quasi-stable state acceleration in SJ-10 was about 1.2 × 10^−6^ G. The grown crystal recovered after the space experiment and cut longitudinally into two pieces along the growth direction. A similar procedure was followed to cut the ground sample and analyses. The indium composition along the longitudinal and radial positions was measured by Electron Probe Micro Analyzer (EPMA) with the detection error of less than 0.002.

### Reporting summary

Further information on experimental design is available in the Nature Research Reporting Summary linked to this article.

## Supplementary information

Reporting Summary

## Data Availability

The datasets generated and analyzed during the current study are available from the corresponding author on reasonable request.

## Supplementary information

**Supplementary information** accompanies the paper on the *npj Microgravity* website (10.1038/s41526-019-0068-1).
